# Supplementary figures and images for: The wide spectrum anti-inflammatory activity of andrographolide in comparison to NSAIDs: A promising therapeutic compound against the cytokine storm
Source: PLoS One. 2024 Jul 17;19(7):e0299965. doi: 10.1371/journal.pone.0299965 (PMC11253928; doi:10.1371/journal.pone.0299965)

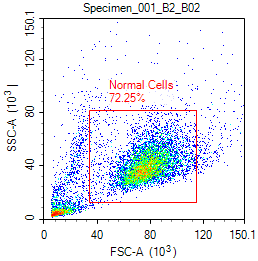

Supplement: S2 File — (ZIP) [file pone.0299965.s002.zip › Supplement No.2 Flow Cytometry/flow/Specimen_001_Andro top dose/Plot1.png]

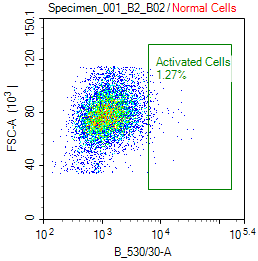

Supplement: S2 File — (ZIP) [file pone.0299965.s002.zip › Supplement No.2 Flow Cytometry/flow/Specimen_001_Andro top dose/Plot2.png]

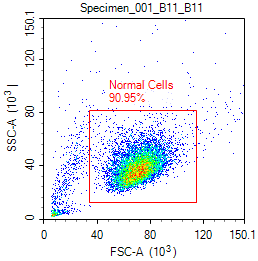

Supplement: S2 File — (ZIP) [file pone.0299965.s002.zip › Supplement No.2 Flow Cytometry/flow/Specimen_001_minus LPS/Plot1.png]

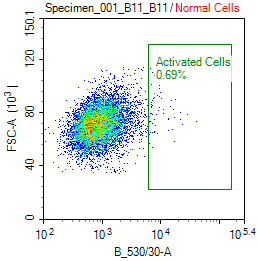

Supplement: S2 File — (ZIP) [file pone.0299965.s002.zip › Supplement No.2 Flow Cytometry/flow/Specimen_001_minus LPS/Plot2.png]

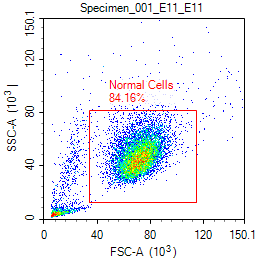

Supplement: S2 File — (ZIP) [file pone.0299965.s002.zip › Supplement No.2 Flow Cytometry/flow/Specimen_001_plus LPS/Plot1.png]

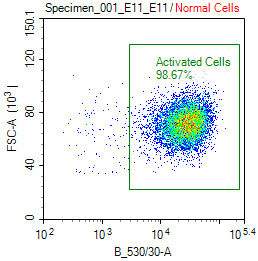

Supplement: S2 File — (ZIP) [file pone.0299965.s002.zip › Supplement No.2 Flow Cytometry/flow/Specimen_001_plus LPS/Plot2.png]
